# Internal Versus External Fixation for the Treatment of Distal Radial Fractures: A Systematic Review of Overlapping Meta-Analyses

**DOI:** 10.1097/MD.0000000000002945

**Published:** 2016-03-07

**Authors:** Qingyu Zhang, Fanxiao Liu, Zhenyun Xiao, Zhenfeng Li, Bomin Wang, Jinlei Dong, Yong Han, Dongsheng Zhou, Jianmin Li

**Affiliations:** From the Department of Orthopedics (QZ, ZL, JL), Qilu Hospital, Shandong University, Jinan, Shandong; Department of Orthopedics (FL, BW, DZ, JD, YH), Shandong Provincial Hospital Affiliated to Shandong University, Jinan, Shandong; and Department of Joint Surgery (ZX), Heze Municipal Hospital, Heze, Shandong, China.

## Abstract

Although a serious of meta-analyses have been published to compare the effects of internal versus external fixation (IF vs EF) for treating distal radial fractures (DRF), no consensus was obtained.

By performing a systematic review of overlapping meta-analyses comparing IF versus EF for the treatment of distal radial fractures, we attempted to evaluate the methodology and reporting quality of these meta-analyses, interpret the source of discordant results, and therefore determine the dominant strategy for the treatment of distal radial fractures based on the best evidence currently.

An electronic databases search was conducted in MEDLINE, Embase, and Cochrane library to retrieve meta-analyses comparing IF versus EF for treating DRF. Reference lists of relevant literatures were also screened manually to retrieve additional ones. Two investigators independently assessed the eligibility of retrieved articles using predefined inclusion and exclusion criteria. All characteristics as well as outcome variables including functional outcomes, range of motion, radiological results, and complication rates with relevant heterogeneity information presented in each included study were extracted. Heterogeneity was thought to be significant when *I*^2^ > 50%. We adopted the Oxford Levels of Evidence and the Assessment of Multiple Systematic Reviews (AMSTAR) Instrument to assess the methodological quality of every included study, and applied the Jadad decision algorithm to select studies with more likely reliable conclusions.

A total of 8 studies met the inclusion criteria. The AMSTAR scores ranged from 5 to 9 with a median of 7.75. Following the Jadad algorithm, the meta-analyses with most reliable results can be selected based on the search strategies and application of selection. Finally, 2 meta-analyses with most RCTs and highest AMSTAR scores were selected in this systematic review of overlapping meta-analysis. The best available evidence suggested that compared with EF, IF was significantly associated with lower Disabilities of the Arm, Shoulder and Hand (DASH) scores, better rehabilitation of volar tilt and radial inclination, and lower infection rate at 1-year follow-up. Therefore, we could conclude that internal fixation is superior to external fixations for the treatment of distal radial fractures.

## INTRODUCTION

Fracture of distal radius (DRF), which accounts for > 8% of all bony injuries in emergency room, is more likely to occur in the pediatric and elderly populations.^1^ DRF could result in permanent impairment and pins; meanwhile, the great absolute number of DRF brings substantial economic costs annually, which is gradually increasing with the aging of population worldwide.^2^ Therefore, choosing effective and evidence-based treatment method is crucial.

Nowadays, treatment choices of DRF are multiple, including internal fixation (IF) with plate (especially volar locking plate [VLP]), cast immobilization, closed reduction with external fixation (EF), as well as percutaneous Kirschner-wire fixation. Although the best choice depends on characteristics of fractures to some extent, IF and EF are 2 most commonly used techniques in recent years. EF could achieve acceptable outcome with less damage. Nevertheless, some studies suggested that recurrent displacements rate was >50% and multiple complications occurred in 20% to 35% patients after EF.^3,4^ More recently, open reduction and internal fixation (ORIF) with VLP was introduced and proved to provide robust and satisfactory stability. It is distinct that unsatisfactory outcomes can occur in both EF and IF;^5–8^ whether one method was superior to the other in clinical outcomes was inconclusive, needing well-designed clinical and biomechanical studies.

Multiple meta-analyses on this topic have been performed in this decade, among them, some only included randomized controlled trials (RCTs) whereas others not. In 2005, Margaliot et al^9^ first published a meta-analysis demonstrating no evidence to support the use of IF over traditional EF. Although several updated meta-analyses supported this result, some refuted it and suggested IF yielded significantly better outcomes. These inconclusive meta-analyses lead to conflicting in clinicians regarding the treatment choice of DRF, which make our systematic review more precise. In order to evaluate the methodology and reporting quality of meta-analyses comparing effects of IF and EF in the treatment of DRF, investigate the source of discordant results, and therefore recommend a best method for treating DRF based on the currently available evidence, we carefully retrieved published meta-analyses on this topic and conducted this systematic review of overlapping meta-analysis.

## METHODS

### Databases Search

MEDLINE, Embase, and Cochrane library were comprehensively searched with the following keywords: (1) “radial” OR “radius” AND (2) “distal” AND (3) “systematic review” OR “meta-analysis.” Reference lists of relevant published literatures were also checked by hands to identify additional eligible meta-analyses. No restrictions of published languages were imposed and the date of last search was July 30, 2015.

### Study Selection

The included studies should meet following criteria: (1) meta-analyses; (2) outcomes of internal fixation versus external fixation for treating distal radial fractures were reported; (3) literatures providing at least 1 variable outcome (e.g., DASH score, range of movements (ROM), grip strength, radiological characteristics, and complication rate); (4) pooled results were calculated. Exclusion criteria included: nonhuman subject; narrative review; systematic review without quantitatively analysis; abstract or conference proceedings due to lack of necessary information and methodology description.

By using aforementioned criteria, 2 investigators (QYZ and FXL) independently and in duplicate performed following study selection process: first, they screened retrieved titles and abstracts to exclude apparently ineligible studies; next, full text of remainders were downloaded and assessed in detail. All discrepancies were resolved though discussion and consensus.

### Data Extraction

All useful information and data were extracted into a standardized excel forms by 2 investigations (QYZ and FXL) independently and checked repeatedly. Main extracted data included surname of first author, year of publication, search databases and date of last search, primary trails design, participants, no of included RCTs, level of evidence, conflicts of interest, and outcome variables (including related heterogeneity information). For meta-analyses included non-RCTs, we excluded those non-RCTs and recalculated results of variables with I-square statistics using modeling and original data provided by those meta-analyses. Another author (ZFL) was consulted to deal with discrepancies.

### Assessment of Methodological Quality and Heterogeneity

The Assessment of Multiple Systematic Reviews (AMSTAR) Instrument^10–12^ is a valuable measurement tool widely used to evaluate the methodological quality of systematic review or meta-analysis. This tool consists of 11 items, each described for 1 score, and higher scores reflect better quality. The Oxford Levels of Evidence^13,14^ is a hierarchy designed as a shortcut for busy clinicians and researchers to find the likely best evidence. Two investigations (QYZ and FXL) independently applied the Oxford Levels of Evidence and the AMSTAR Instrument to evaluate methodological quality of each included literature. All disagreements were discussed to reach a consensus.

*I*-square statistic is a quantitative measure describing the percentage of total variation due to heterogeneity. The extracted *I*-square statistic value was used to assess the heterogeneity of each variable across studies. According to the Cochrane Handbook, between-study heterogeneity of variables is considered to be substantial when the *I*-square range from 50% to 90%. Therefore, an *I*-square of <50% is acceptable in this systematic review. For variables with significant between-study heterogeneity, whether the included meta-analyses explored the source of heterogeneity was recorded. Meanwhile, we also recorded whether these studies performed sensitivity analysis and evaluated publication bias.

### Application of Jadad Decision Algorithm

Treatment recommendations were further determined by using the Jadad decision algorithm,^15^ which explore the discordance between systematic reviews and meta-analyses including differences in question proposal, selection criteria, data extraction, heterogeneity testing, data synthesis, trial quality, search strategy, and so on. Same as before, this tool was applied by 2 investigators independently to determine which meta-analyses provided the best currently available evidence and any discrepancies were resolved by discussion and consensus.

In this investigation, “guidelines of Preferred Reporting Items for Systematic Reviews and Meta-analysis”^16^ was followed to ensure reporting quality. Meanwhile, as all data were extracted from published meta-analyses, ethical approval and informed patient consent were not required.

## RESULTS

### Study Selection and Characteristics

**FIGURE 1:**
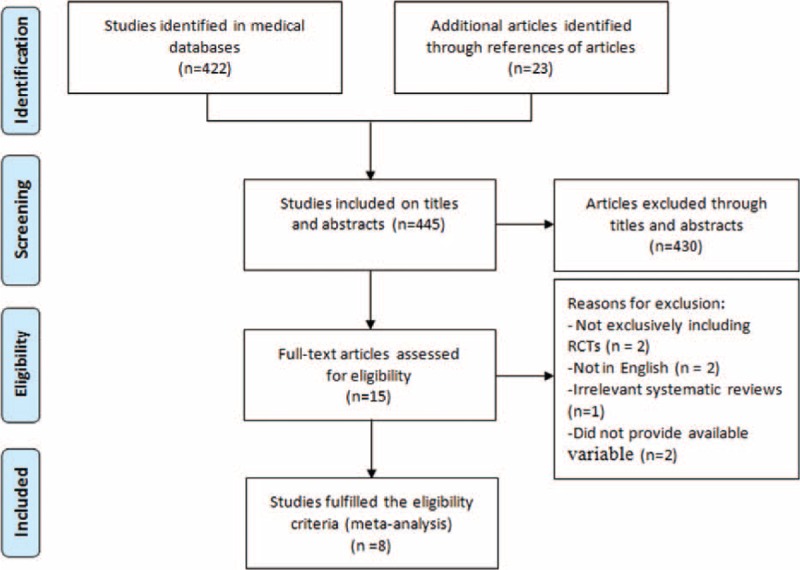
Flowchart summarizing the selection process of meta-analyses.

Initially, 445 articles were retrieved by the search of 3 electronic databases and references of relevant articles. By screening titles and abstracts, 430 apparently irrelevant articles were first excluded. Then, the full texts of remainders were downloaded to assess in detail. Eventually, eight^17–24^ meta-analyses published between 2011 and 2015 were included in this systematic review. The search process and exclusion reasons were described in detail in Figure 1. The number of included primary RCTs of these meta-analyses ranged widely from 3 to 11.^25–42^ The basic information of each included literature can be found in Tables 1 and 2.

**TABLE 1:**
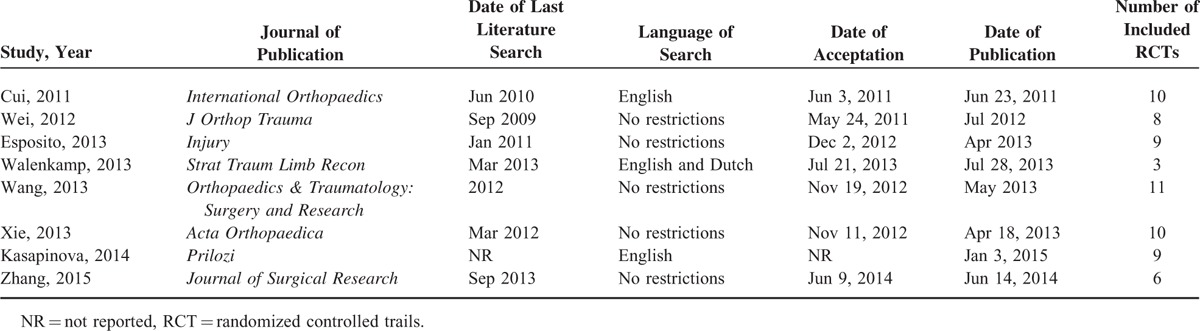
General Description of the Characteristics of Each Meta-Analysis

**TABLE 2:**
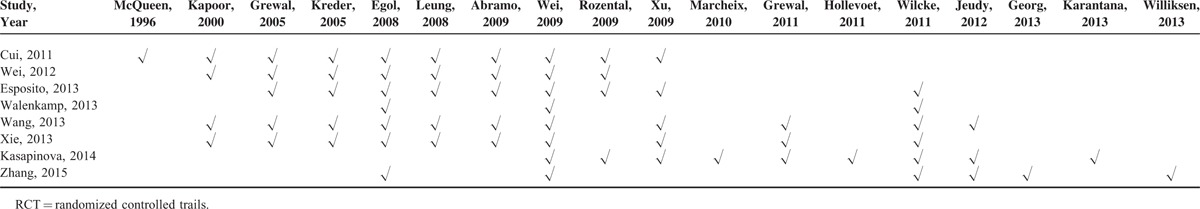
Primary RCTs Included in Meta-Analyses

### Search Strategy Assessment

All 8 included meta-analyses searched MEDLINE and among them, seven^17–21,23,24^ declared that they also searched Embase and Cochrane Library. Heterogeneity existed as to whether studies searched OVID, Web of Knowledge, BIOSIS, and SCOPUS. Details of search methodology used by each included meta-analysis were presented in Table 3.

**TABLE 3:**
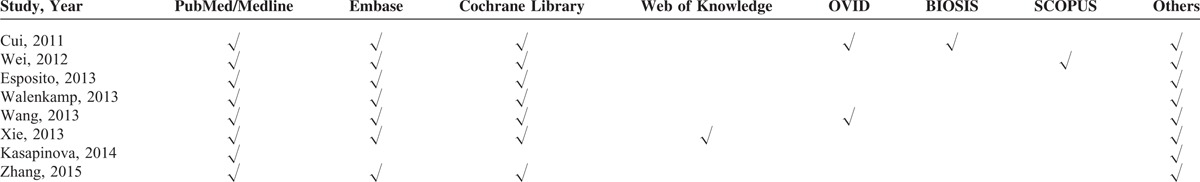
Databases Used by Each Meta-Analysis in their Study Searches

### Methodological Quality Assessment

Among included meta-analyses, 6 only included RCTs; the other two^18,23^ included 1 and 4 non-RCTs, respectively. For latter 2 studies, we excluded those non-RCTs and recalculated relevant variables with *I*-square statistics using provided modeling. Therefore all studies were level II evidence. Two^17,21^ studies reported that GRADE was used in their research and four^17,20,21,24^ studies declared to conduct investigations following PRISMA statement (Table 4). The revised methodological information was listed in Table 4 in detail. AMSTAR scores of included 8 studies varied from 6 to 9, one^18^ study receiving 5 scores, one^21^ receiving 7, four^17,22–24^ receiving 8, and two^19,20^ meta-analyses met 9 items of the 11 (82%) of the AMSTAR criteria receiving 9 scores (Table 5).

**TABLE 4:**
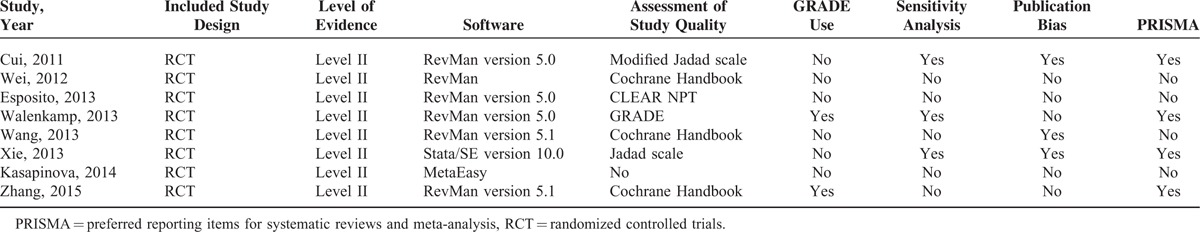
Methodological Information for Each Included Meta-Analysis

**TABLE 5:**
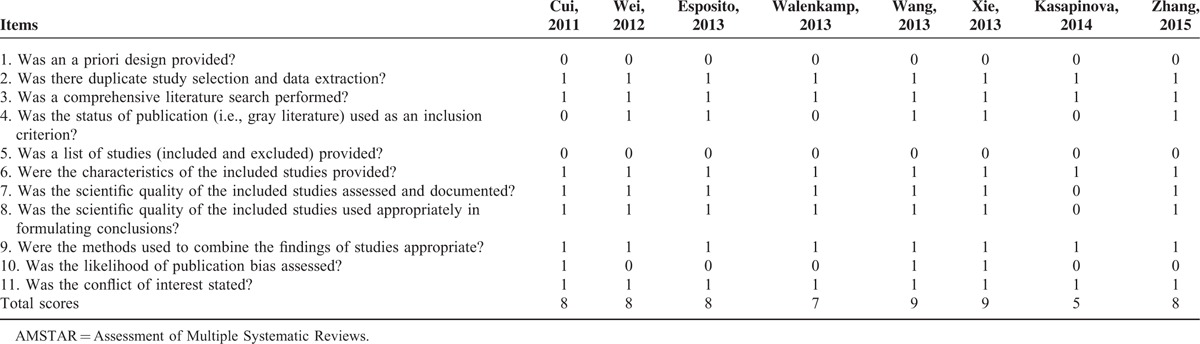
AMSTAR Criteria for Each Included Meta-Analysis

### Heterogeneity Assessment

All extracted variables provided related *I*-square statistic information (Table 6). Of the 8 meta-analyses, three^20,21,24^ meta-analyses conducted sensitivity analyses based on publication status or methodological quality (Table 4).

**TABLE 6:**
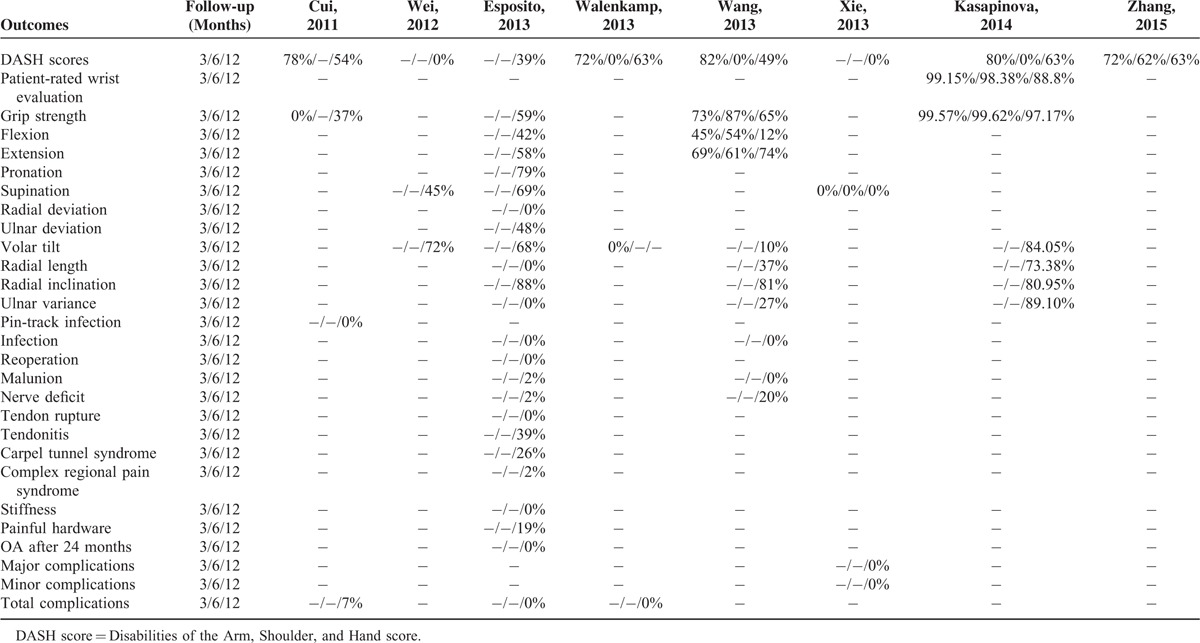
I-Square Statistic Value of Each Variable at 3, 6, and 12 Months Follow-Up in Each Meta-Analysis

### Results of Jadad Decision Algorithm

**FIGURE 2:**
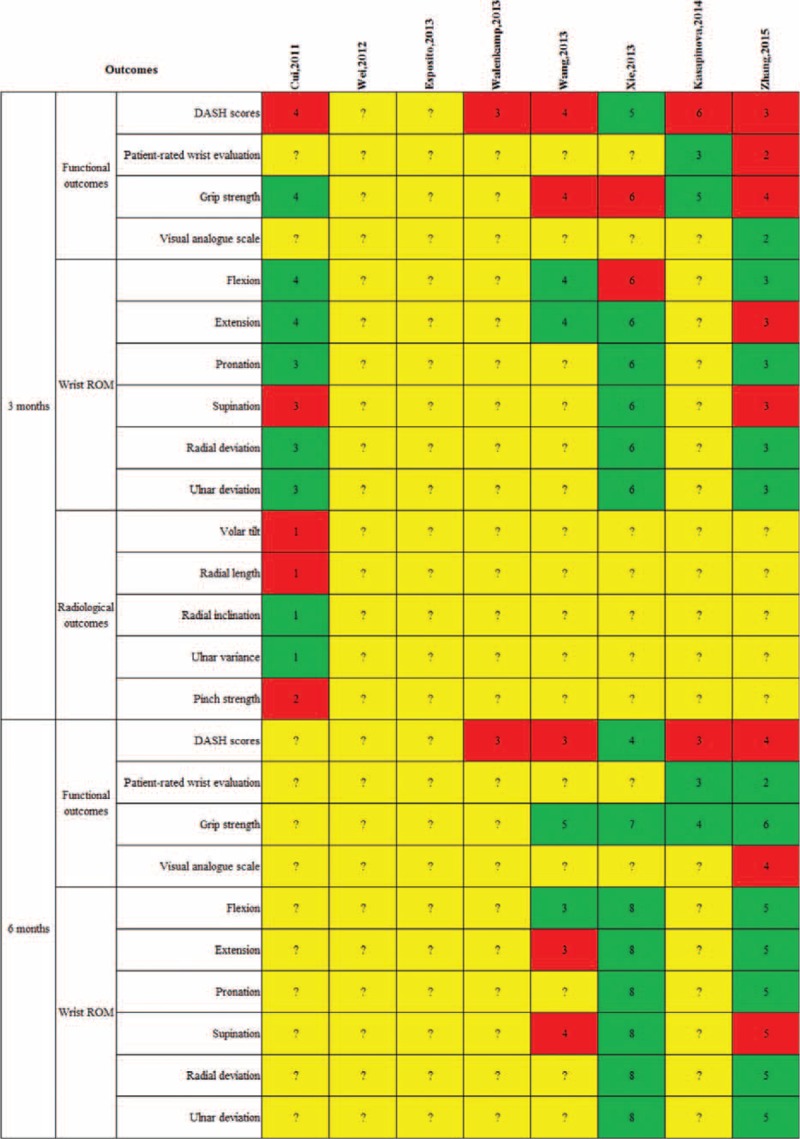
Outcomes of each included meta-analysis at 3 and 6 months follow-up. Red means favoring plate; green means no difference; yellow means not reporting; and blue means favoring nail. Arabic numerals mean the number of included randomized clinical trials.

**FIGURE 3:**
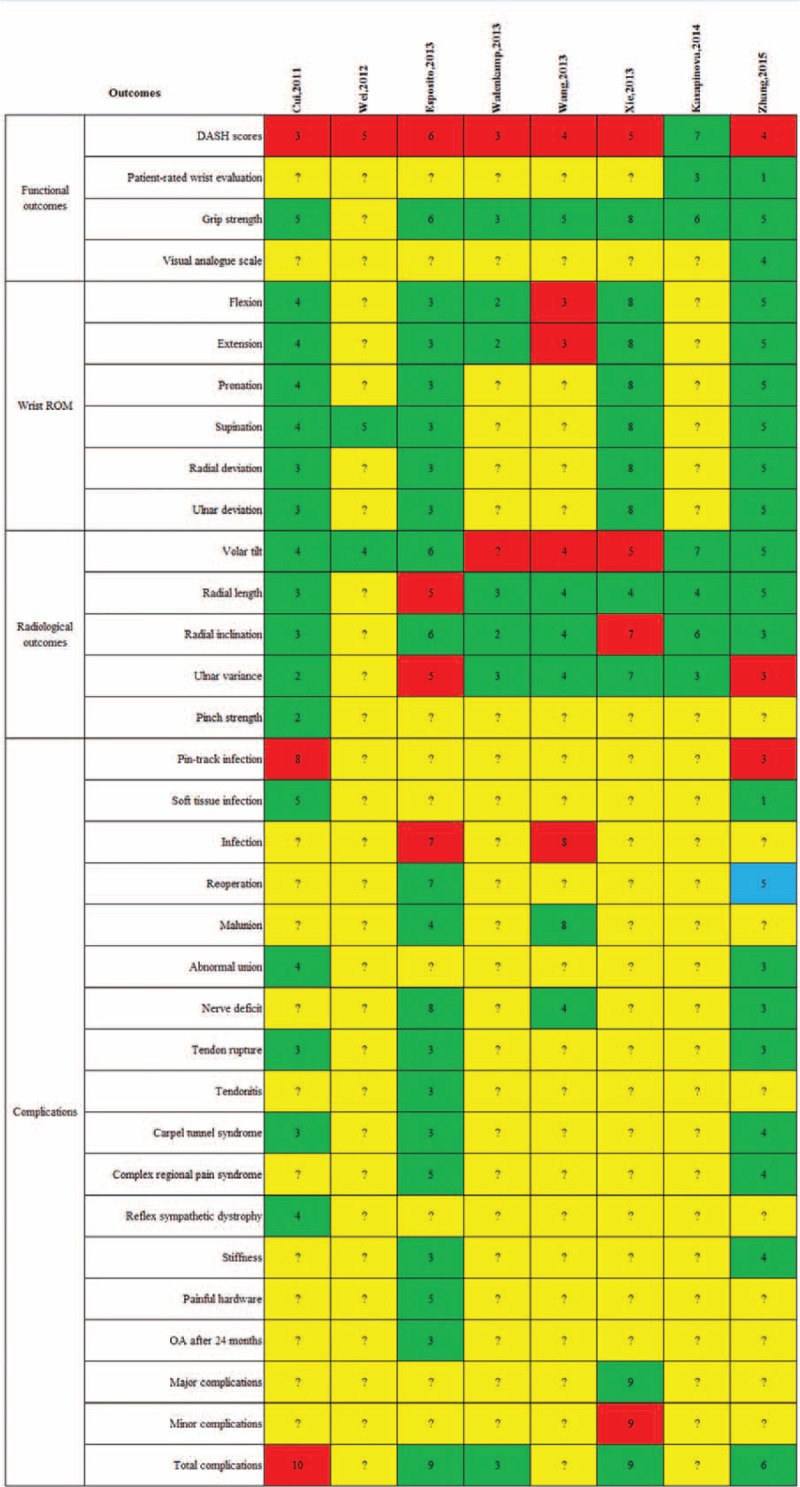
Outcomes of each included meta-analysis at 12 months follow-up. Red means favoring plate; green means no difference; yellow means not reporting; and blue means favoring nail. Arabic numerals mean the number of included randomized clinical trials.

**FIGURE 4:**
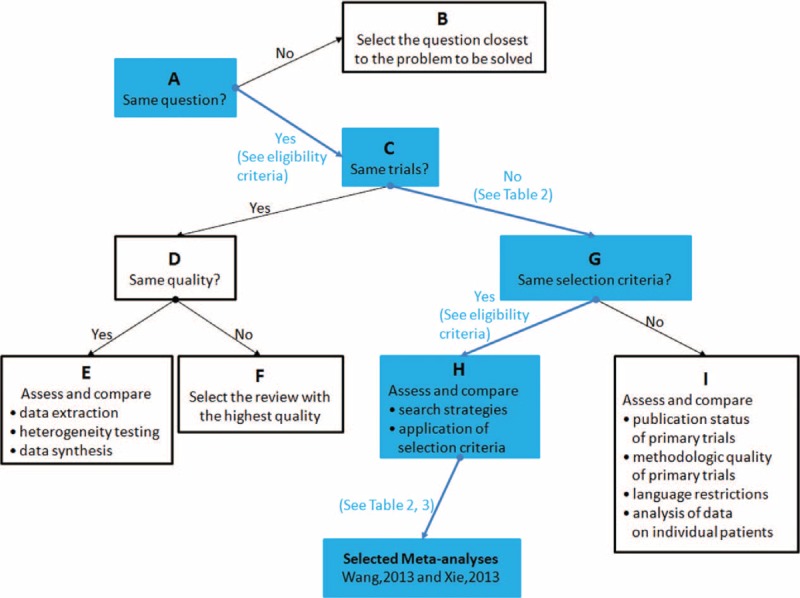
Flowchart of Jadad decision algorithm.

Outcomes of meta-analyses included in this systematic review were described in Figures 2 and 3. Two investigators (QYZ and FXL) carefully read these articles and confirmed that they all targeted at the same clinical question. These meta-analyses enroled different primary trials with similar selection criteria. Therefore, according to the Jadad algorithm, the best available evidences should be assessed and compared on the basis of search strategies and application of selection criteria. Eventually, 2 meta-analyses including more RCTs performed by Wang et al^19^ in 2013 and Xie et al^20^ in 2013 respectively were selected (Figure 4).

### Results of Best Available Evidence

These 2 meta-analyses selected by the Jadad Decision Algorithm also had the highest AMSTAR scores in all included studies. They all compared 2 methods for treating DRF on 4 aspects including functional outcomes, range of motion (ROM), radiological outcomes, and complication rates. The best available evidence currently suggested that compared with EF, IF was significantly associated with lower DASH scores, better rehabilitation of volar tilt and radial inclination, and lower infection rate at 1 year follow-up (Figures 2 and 3).

## DISCUSSION

Although well-designed meta-analyses of randomized controlled trials (RCTs) could provide highest evidence for clinical decision-making, overlapping ones with discordant results may also bring misleading to patients and surgeons. As mentioned above, DRF is a very common type of fracture and nowadays, IF and external fixation (EF) are both widely used in clinic. A large amount of trials, among them, some were RCTs whereas some were not, investigated the difference of IF versus EF in the treatment of distal radial fractures; however, no consensus were reached. Therefore, more recently, a series of overlapping meta-analyses were conducted to further explore this issue by pooling relevant studies. Unfortunately, homogenous conclusion was still unavailable. Up to now, with regard to the evidence for the treatment opinions of DRF, the recommendation summary of the American Academy of Orthopedic Surgeons clinical practice guideline was “inconclusive.”^43^

In this investigation, the comprehensive study searching and selection yielded a total of 8 meta-analyses on this topic. Among included studies, five^19–22,24^ concluded that IF is superior to EF in the treatment of DRF, two^17,18^ did not demonstrate obvious advantage of either of the 2 fixation methods; although some benefits of EF over IF were suggested in the meta-analysis conducted by Wei et al^23^ in 2012, they were all negated after the non-RCTs were excluded. Following the decision tool provided by Jadad et al, we selected 2 meta-analysis performed by Xie et al^20^ in 2013 and Wang et al^19^ in 2013, respectively. Meanwhile, these 2 also possessed the highest methodological quality among 8 included overlapping meta-analyses according to the AMSTAR tool. Because all meta-analyses pooled RCTs, they were considered to be level II evidence. Based on currently available best evidence, we could conclude that more advantages could be obtained by using IF in the treatment of DRF. Multiple valuable parameters including functional outcomes, range of motion, radiological results, and complication rates of these 2 methods were assessed in detail.

Main functional outcomes used in these studies including Disabilities of the Arm, Shoulder and Hand (DASH) score and grip strength. DASH score is a self-reported questionnaire used to assess upper extremity function ranging from 0 point (no disability) to 100 points (maximum disability).^44^ All included meta-analyses used DASH scores as the primary outcome. Except the 2 selected meta-analyses, there were another five^17,21–24^ ones revealed lower DASH scores obtained in the IF group at 1 year follow-up. Meanwhile, Wang et al^19^ also reported better DASH scores at 3 months and 6 months follow-up in the IF group, and after excluding patients who did not use VLP, the results were even more favorable. One most possible explanation for this difference of DASH scores is that plate osteosynthesis could better restore the bony anatomy as a stable internal fixation and therefore allow patients to have an early and active mobilization regimen. No included meta-analyses showed difference of IF and EF in the rehabilitation of grip strength.

The main purpose of treating DRF is to obtain a painless wrist with a satisfactory degree of mobility. Therefore, another important index to assess the effect of IF and EF is the ROM, including flexion, extension, pronation, supinaion, radial deviation, and ulnar deviation. The meta-analysis conducted by Wang et al^19^ demonstrated better extension and flexion in IF group compared with those in the EF group. However, these investigations only pooled 3 RCTs in this subgroup analysis. Xie et al^20^ combined 8 RCTs and found no significant advantage of either method in extension and flexion, which was supported by other five^17,21–24^ meta-analyses. No difference between IF and EF in other variables was found according to selected meta-analyses.

Next aspect of rehabilitation of DRF meriting analysis is the radiological outcomes. Both 2 selected meta-analyses demonstrated better volar tilt obtained in the IF group at 1 year follow-up. Wei et al^23^ also suggested favorable radial inclination observed in the IF group, which was demonstrated by the subgroup-analysis based on 7 RCTs. There were no statistical difference in radial length, ulnar variance, and pinch strength.

The last but not least, complication rates are of great importance. Infection is a common complication after EF. In this systematic review, two^19,22^ of 8 enrolled meta-analyses, including a high-quality meta-analysis selected by the Jadad tool, investigated infection after IF and EF and both suggested that the higher infection rate occurred in EF. No difference between 2 methods in other complications was found by 2 selected meta-analyses. Most infection cases in the EF group might be explained by less or incorrect nursing, whereas some deep infection could be ascribed to insufficient sterilization of pins and fixators. In addition, Wang et al^19^ noticed an obviously lower malunion rate in the IF group. However, the difference did not have statistical significance, which warrants further investigation.

For fractures that could not obtain satisfactory function, reduction, or prognosis with nonoperative treatment, surgery is necessary. It is well-known that there were several subtypes of distal radial fractures and internal fixation also includes different techniques (volar/dorsal, locking/nonlocking plate, the TriMed system, etc., open/closed reduction); meanwhile, a majority of the RCTs included in the best available evidences used bridging fixator ± suppl. K-wires and others did not report the methods of external fixation. Therefore, although our study demonstrates that on the whole internal fixation is superior to external fixation in many aspects, the indications and complications of each technique may possess differences. Nowadays, VLPs rank as the most commonly-used option of internal fixation for displaced DRFs and have been proven to be efficient.^19,20,45–47^ Nevertheless, VLPs are no panacea; some surgeons support that for extra-articular DRFs without metaphyseal comminution, closed reduction and percutaneous K-wire fixation are preferable.^45^ Therefore, the optimum treatment strategy should be determined by the characteristics of fractures (age, open or closed, nondisplaced or displaced, extra- or intra-articular, and so on) and the experience of the surgeons. The guidelines of American Academy of Orthopaedic Surgeons (AAOS) presented 29 recommendations for the treatment of DRFs and all of them were weak evidences.^48^ It is notable that percutaneous K-wire fixation is becoming increasingly popular and 1 meta-analysis suggested that IF with VLPs was associated with better DASH scores when compared with percutaneous K-wires for treating dorsally displaced DRFs in adults.^46^ However, another meta-analysis pointed that the difference was small and clinically unimportant.^49^ Therefore, further study exploring these 2 methods is still necessary.

There were some limitations in this investigation merited consideration. First, this is a systematic review of published overlapping meta-analyses, we could only analyz issue on the meta-analysis level, instead of patients or trial level; second, although we only included the meta-analyses exclusively pooling RCTs to ensure the quality of our investigation, all meta-analyses was level II of evidence. Last but not least, some meta-analyses included and analyzed lower-quality RCTs.

## CONCLUSION

By systematically assessing overlapping meta-analyses comparing internal fixation versus external fixation for the treatment of distal radial fracture, the selected best evidence suggested that compared with EF, IF was significantly associated with lower DASH scores, better rehabilitation of volar tilt and radial inclination, and lower infection rates at 1 year postoperatively. Therefore, we could safely reach the conclusion that internal fixation is superior to external fixations for the treatment of distal radial fractures. However, further investigations are still needed to warrant current conclusions.
